# The increased in vivo firing of pyramidal cells but not interneurons in the anterior cingulate cortex after neuropathic pain

**DOI:** 10.1186/s13041-022-00897-9

**Published:** 2022-01-29

**Authors:** Da-Yu Zhu, Ting-Ting Cao, Hong-Wei Fan, Ming-Zhe Zhang, Hao-Kai Duan, Jing Li, Xia-Jing Zhang, Yun-Qing Li, Pan Wang, Tao Chen

**Affiliations:** 1 Department of Human Anatomy, Xuzhou Medical University, Xuzhou, 221004 Jiangsu China; 2 Department of Human Anatomy, Histology and Embryology & K.K. Leung Brain Research Centre, The Fourth Military Medical University, No. 169 Changle West Road, Xi’an, 710032 China; 3 Institute of Medical Research, Northwestern Polytechnical University, Xi’an, 710072 Shaanxi China; 4 Department of Psychology, Institute of Public Health, Xi’an Medical University, Xi’an, 710021 China

**Keywords:** Anterior cingulate cortex, Neuropathic pain, Pyramidal cell, Interneuron, In vivo multi-channel recording, Mice

## Abstract

Chronic pain damages the balance between excitation and inhibition in the sensory cortex. It has been confirmed that the activity of cortical glutamatergic pyramidal cells increases after chronic pain. However, whether the activity of inhibitory interneurons synchronized changed remains obscure, especially in in vivo conditions. In the present study, we checked the firing rate of pyramidal cells and interneurons in the anterior cingulate cortex, a main cortical area for the regulation of nociceptive information in mice with spared nerve injury by using in vivo multi-channel recording system. We found that the firing rate of pyramidal cells but not interneurons increased in the ACC, which was further confirmed by the increased FOS expression in pyramidal cells but not interneurons, in mice with neuropathic pain. Selectively high frequency stimulation of the ACC nociceptive afferent fibers only potentiated the activity of pyramidal cells either. Our results thus suggest that the increased activity of pyramidal cells contributes to the damaged E/I balance in the ACC and is important for the pain hypersensitivity in mice with neuropathic pain.

## Introduction

Neuropathic pain (NP) is a chronic disease caused by a primary disease or lesion affecting the somatosensory nervous system [1, 2]. It is well documented that anterior cingulate cortex (ACC), a forebrain structure, plays an important role for the process and regulation of neuropathic pain [3, 4]. ACC can be activated in acute pain or NP conditions [5], while chemical inhibition or destruction of ACC produces analgesic effects [6].

In cortex, excitatory pyramidal neurons (70–80% of the total number of neurons) release glutamate, while the inhibitory interneurons (20–30% of the total number of neurons) release gamma-aminobutyric acid (GABA) in the neural cortical network [7, 8]. In the ACC, it’s reported that selective activation of pyramidal neurons by using optogenetic methods decreases pain thresholds rapidly and induces allodynia, and specific inhibition of pyramidal neurons or activation of interneurons induces analgesic effects in mice [3, 9]. These results together indicate that the excitatory or inhibitory neuronal activity in the ACC significantly affect nociception and pain responses in mice.

However, how does the activity of cortical pyramidal cells and interneurons change in the condition of chronic pain remains controversial. Some report that the activities of pyramidal cells and interneurons are synchronized increased after inflammatory chronic pain [10]. While others indicate that chronic pain induces increased activity of pyramidal cells but decreased activity of interneurons in mice [11–13]. However, those reported studies are investigated by using Ca^2+^-imaging or in vitro electrophysiological recording methods, it is thus necessary to directly identify the electrophysiological activities of cortical pyramidal cells and interneurons in in vivo condition and to evaluate the balance between excitatory and inhibitory neurons after chronic pain.

In the present study, we checked the spike of pyramidal cells and interneurons in the ACC in mice with chronic neuropathic pain, by using in vivo multichannel electrophysiology recording techniques. Our study showed that the firing rate of pyramidal cells increased significantly in mice after nerve injury. However, the firing rate of interneurons remained unchanged. When delivering optical high frequency stimulation (oHFS) to the ACC-afferent fibers expressing channelrhodopsin-2 (ChR2), only the firing rate of pyramidal cells but not the interneurons increased in both sham operated and nerve injured mice. Our data show that the in vivo increased spike of pyramidal cells contributes to the damaged cortical E/I balance, which may be important for the enhanced pain responses in mice with neuropathic pain.

## Results

### Spared nerve injury induced significant pain hypersensitivity in mice

**Fig. 1:**
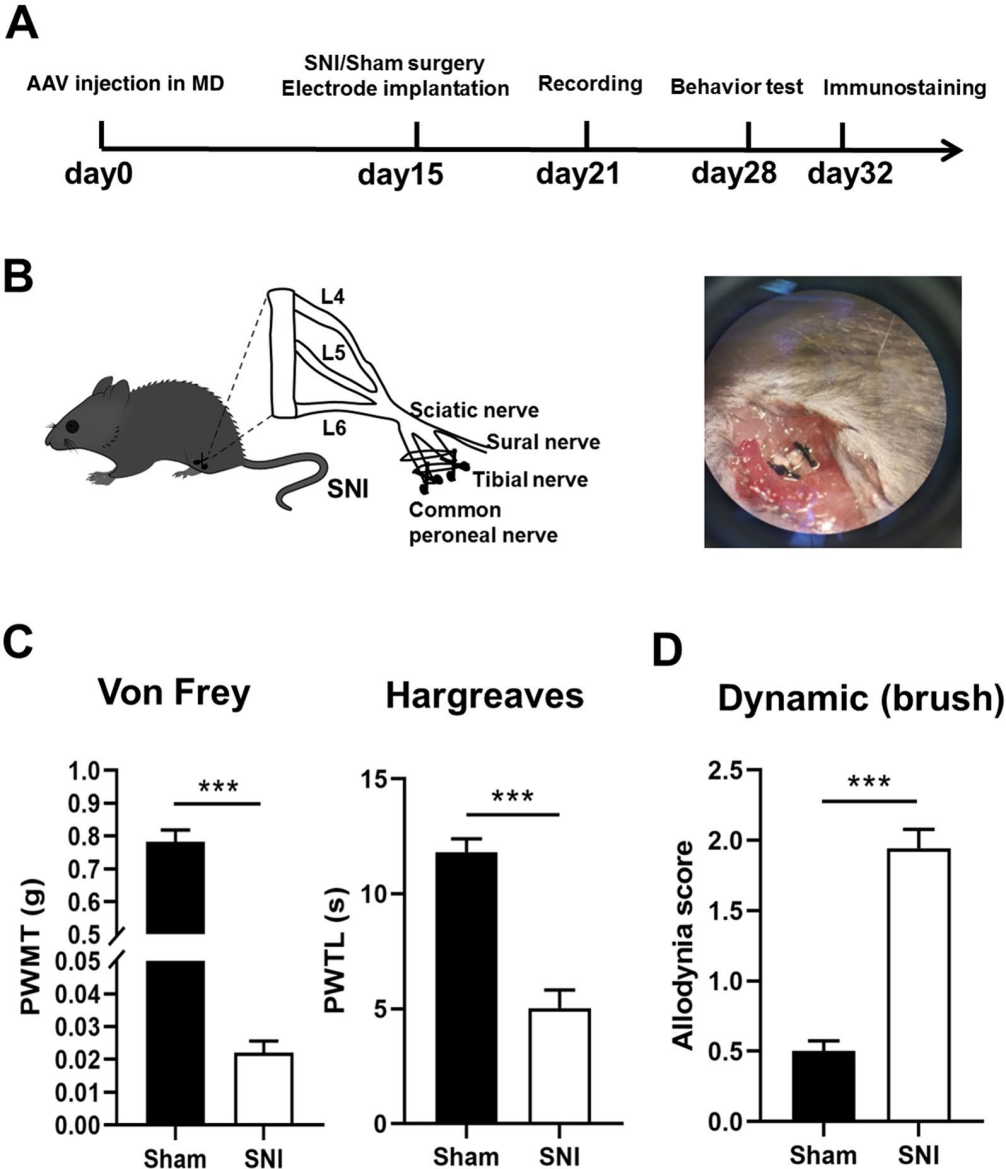
SNI induces pain hypersensitivity in mice. **A** Experimental timeline. **B** Schematic of the spared nerve injury (SNI) surgery. The tibial nerve and common peroneal nerve are ligated and cut, and the sural nerve is retained. **C** The PWMT and PWTL are significantly decreased in SNI mice in comparison with sham-operated mice. ***p < 0.001 (n = 8 mice in each group, unpaired *t*-test). **D** The spontaneous pain score is significantly increased in SNI mice in comparison with sham-operated mice. ***p < 0.001 (n = 8 mice in each group, unpaired *t*-test)

To access neuropathic pain, spared nerve injury (SNI) model was established on adult mice on Day 0 (Fig. 1A, B). The possible static and dynamic pain responses were then evaluated by measuring the paw withdrawal mechanical thresholds (PWMT) in *von frey* filament test, paw withdrawal thermal latency (PWTL) in Hargreaves test, and spontaneous allodynia score by stroking the hindpaw plantar surface with a soft paintbrush. We observed that the PWMT and PWTL were significantly decreased, and the allodynia score was significantly increased in SNI group when compared to the sham group at day 7 post-surgery (Fig. 1C, D). These behavioral tests results reveal that obvious mechanical allodynia, heat hyperalgesia and spontaneous pain are induced in SNI mice.

### Spared nerve injury increased FOS expression in pyramidal cells but not interneurons

**Fig. 2:**
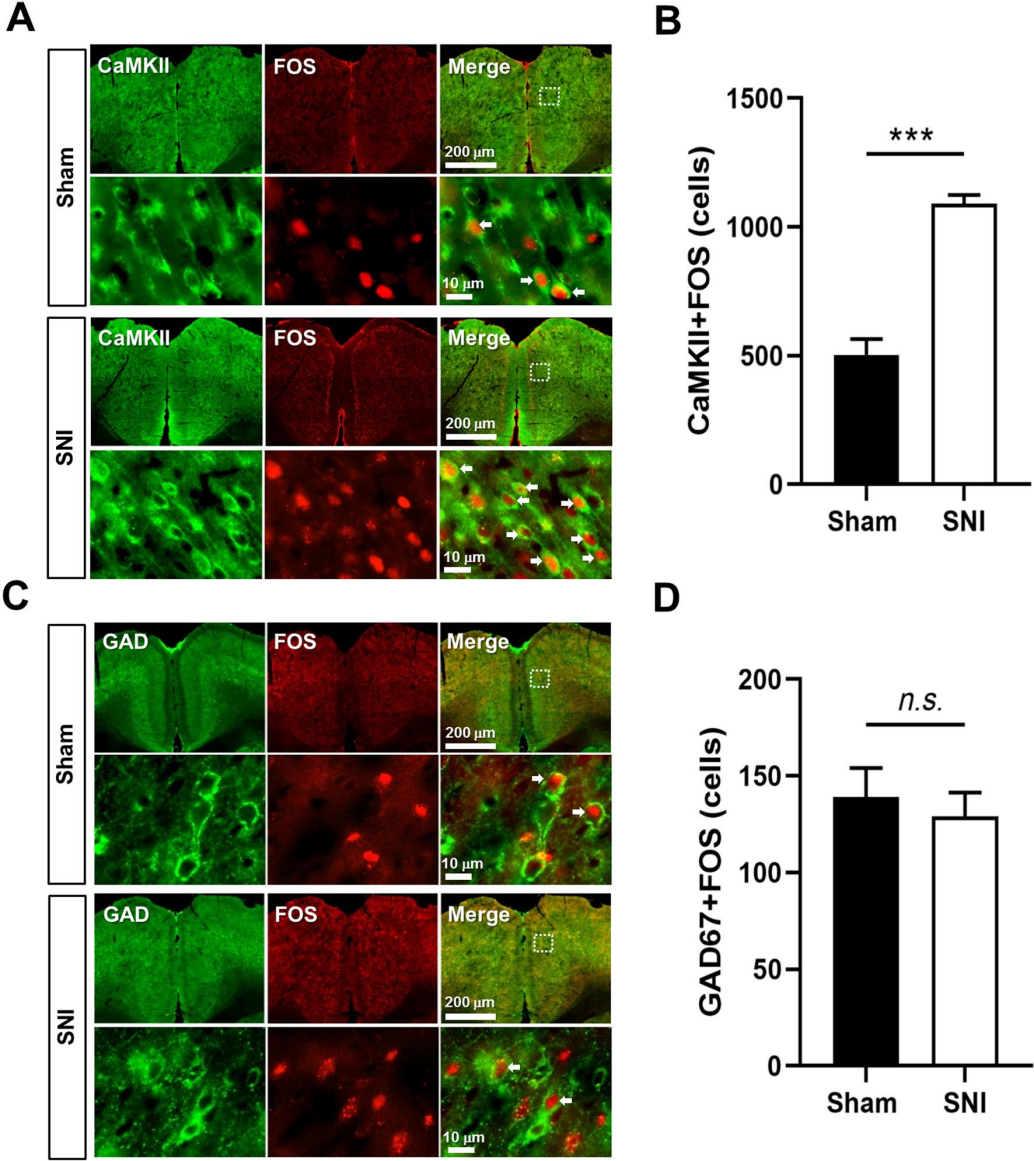
The increased FOS expression in pyramidal cells but not interneurons in the ACC of SNI mice. **A** Representative immunofluorescent figures of CaMKII-immunoreactive (green) and FOS-immunoreactive (red) neurons in the ACC of sham and SNI mice. The magnified images of the dotted rectangle areas are shown in the lower panels respectively. **B** The average number of CaMKII/FOS double-labeling neurons. **C** Representative immunofluorescent figures of GAD67-immunoreactive (green) and FOS-immunoreactive (red) neurons in the ACC of sham and SNI mice. The magnified images of the dotted rectangle areas are shown in the lower panels respectively. **D** The average number of GAD67/FOS double-labeling neurons. ***p < 0.001, *n.s.* not significant (n = 4 mice in each group, unpaired *t*-test)

Accumulating evidences show the involvement of ACC in the regulation of pain [3], we thus feel interest to check whether the ACC pyramidal cells and interneurons were simultaneously activated in SNI mice 7 days post-surgery. The expression of FOS protein, an activity-dependent neuronal marker, was then investigated in the pyramidal cells and interneurons in the ACC. By using double-immunofluorescent staining of CaMKII or GAD67 with FOS, we found that the number of FOS/CaMKII double-labeled (FOS/CaMKII^+^) neurons was significantly increased in SNI group in comparison with that in the sham group (Fig. 2A, B). However, the number of FOS/GAD67 double-labeled (FOS/GAD67^+^) neurons was not different between SNI and sham groups (Fig. 2C, D). The results suggest that the activities of pyramidal cells but not interneurons are increased in the ACC in mice with neuropathic pain.

### Spared nerve injury increased the excitability of pyramidal cells in the ACC

**Fig. 3:**
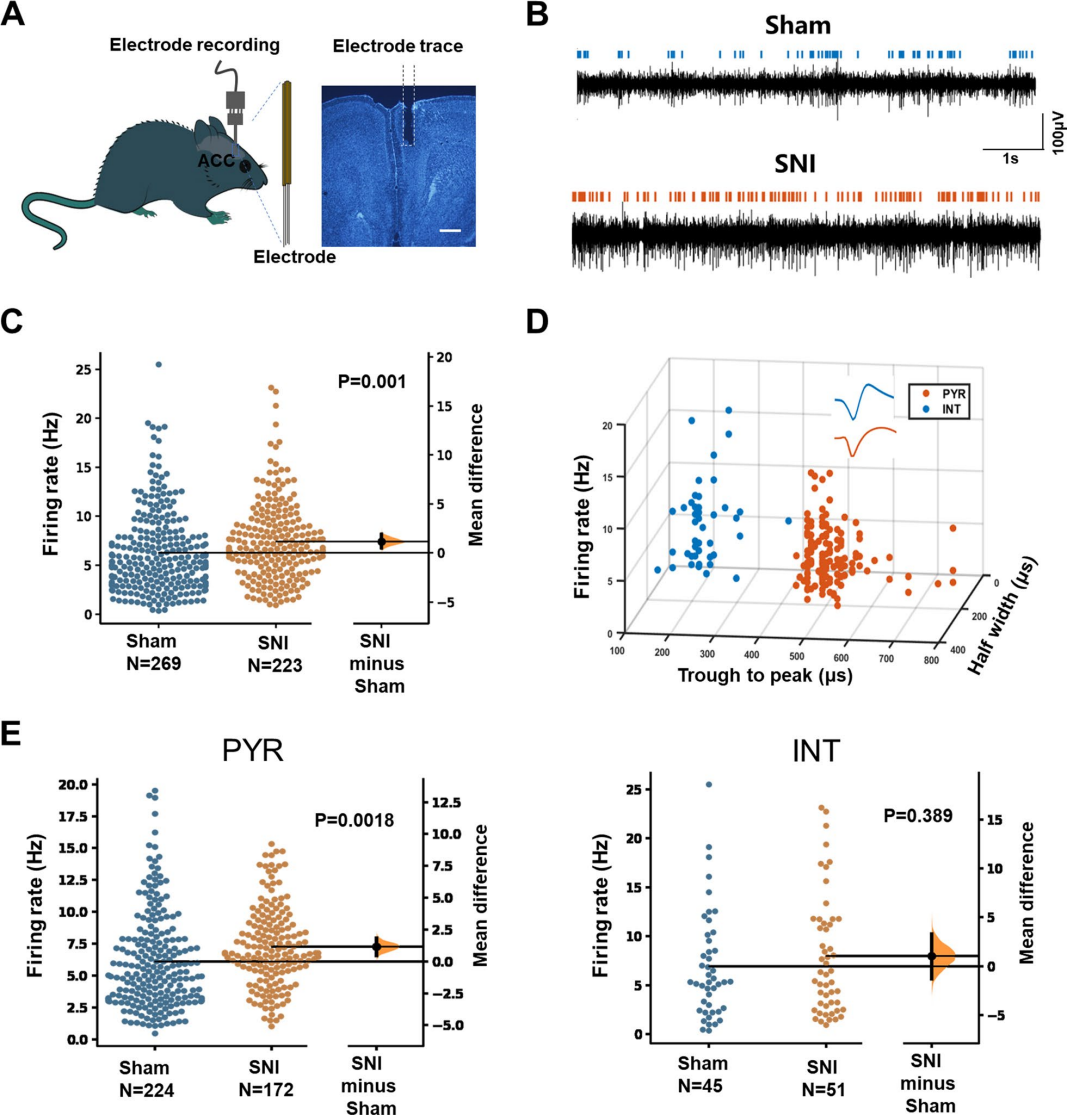
The increased firing rate of pyramidal cells in the ACC of SNI mice. **A** Schematic showing the single-unit recording of ACC in awake mice using in vivo multi-channel recording technique. One example image on the right panel showing the electrodes trace in the ACC. Scale bar = 500 μm. **B** Example recording signals of ACC neurons. The blue or orange lines indicate the spikes in one sham-operated or one SNI mouse, respectively. **C** The firing rates of neurons in sham and SNI groups are plotted by using the Gardner-Altman estimation method [31]. Both groups are plotted on the left axes; the mean difference is plotted on the floating axes on the right as a bootstrap sampling distribution. The mean difference is depicted as a dot and the 95% confidence interval is indicated by the ends of the vertical error bar. *p* = 0.001 (n = 3 mice in sham and SNI group, paired t-test). **D** Partial recorded ACC neurons (n = 194) were classified as pyramidal cells (149) and interneurons (45) using k-means cluster-separation algorithm based on their electrophysiological properties. Each dot represents one cell. Inset, the average waveform of a representative pyramidal cell (PYR, red) and interneuron (INT, blue). **E** The firing rates of pyramidal cells (*p* = 0.0018) and interneurons (*p* = 0.389) in sham and SNI groups (paired *t*-test). *PYR* pyramidal cells; *INT* interneurons

We then explored whether the activities of pyramidal cells and interneurons in the ACC were changed in head-fixed awake mice after nerve injury. To this end, we implanted a 16-channel single-unit electrode in the ACC (Fig. 3A) and the activities of ACC neurons were monitored in sham and SNI groups (Fig. 3B). We found that the mean frequency of well-isolated neurons was significantly increased in SNI group (Fig. 3C), indicating an increased activity of ACC neurons.

We want to further explore whether the activities of pyramidal cells and interneurons in the ACC were both changed in SNI mice. The two types of neurons were then identified and classified based on their firing rate, trough-to-peak duration and half width (Fig. 3D), according to the standard array of neuronal properties reported previously [14]. We found that, compared with sham group, the firing rate of pyramidal neurons increased significantly in SNI mice. However, the firing rate of interneurons remained unchanged (Fig. 3E). These results clearly show that only the activities of pyramidal cells but not interneurons increased in the ACC of mice with chronic neuropathic pain.

### High frequency stimulation of ACC afferent fibers increased the activities of pyramidal cells in the ACC

**Fig. 4:**
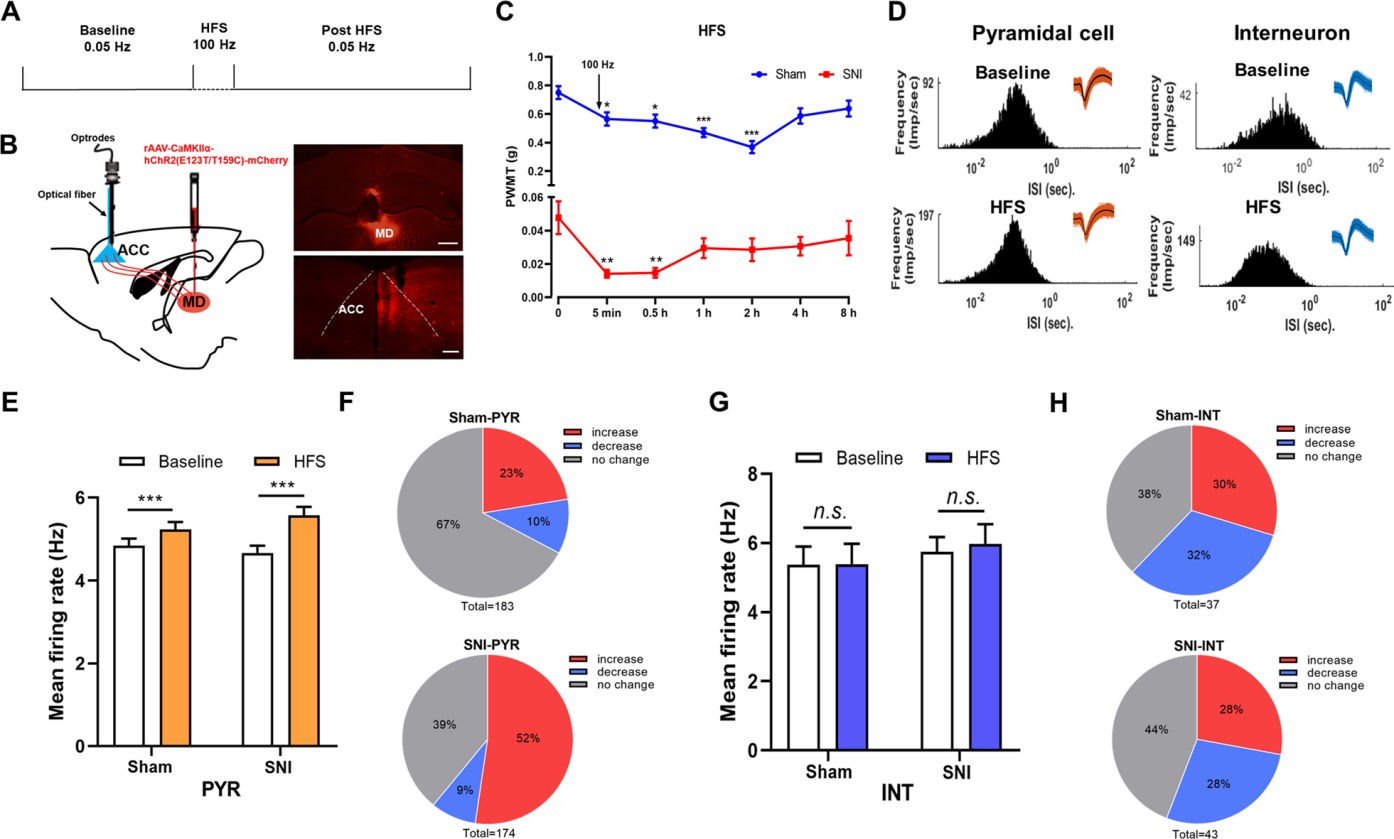
Optical HFS induction of ACC afferent fibers increases the activities of ACC pyramidal cells and induces pain hypersensitivity. **A** Experimental timeline. **B** Schematic showing that, with the injection of rAAV-CaMKIIα-ChR2 (E123T/T159C)-mCherry into the ipsilateral mediodorsal thalamic nucleus (MD), an optic fiber was implanted into the ACC through the multi-channel recording system and the in vivo spikes of ACC neurons are recorded. The coronal sections of virus injection site in the MD and mCherry^+^ projecting fibers in the ACC are shown on the right panel. Scale bars = 500 μm. **C** PWMT before (0 h) and after oHFS induction in sham and SNI mice. *p < 0.05; **p < 0.01; ***p < 0.001, compared with baseline (0 h) (n = 8 mice in each group, One Way ANOVA with Dunnett post hoc analysis). **D** Histograms of the inter-spike intervals (ISI) from the spikes of a pyramidal cell and an interneuron in baseline and post-HFS recording period. Insets at the top right corner show the waveforms of the detected single unit. **E** The averaged firing rate of pyramidal cells (PYR) in sham and SNI group before and after oHFS induction. ***p < 0.001, n = 3 mice in sham and SNI group, paired *t*-test. **F** Proportion of pyramidal cells with changed firing rate in sham and SNI groups. Pie charts summarize the changes in firing rate of pyramidal cells in sham (n = 183 neurons) or SNI (n = 174 neurons) groups. Pre vs. post oHFS induction, Wilcoxon rank-sum test. **G** The averaged firing rate of interneurons (INT) in sham and SNI group before and after oHFS induction. *n.s.* not significant, n = 3 mice in sham and SNI group, paired *t*-test. **H** Proportion of interneurons with changed firing rate in sham and SNI groups. Pie charts summarize the changes in firing rate of interneurons in sham (n = 37 neurons) or SNI (n = 43 neurons) groups. Pre *vs.* post oHFS induction, Wilcoxon rank-sum test

Neuropathic pain induces synaptic long-term potentiation (LTP) in the ACC and LTP induction in the ACC leads to hypersensitivity of pain sensation [3]. We then feel interested to test whether LTP induction could increase the activities of pyramidal cells and interneurons in the ACC (Fig. 4A). Since ipsilateral mediodorsal thalamic nucleus (MD) is the main source of ACC nociceptive afferent fibers [15, 16], we injected rAAV-CaMKIIα-hChR2(E123T/T159C)-mCherry into the MD to label the MD-ACC projecting fibers (Fig. 4B). Optic (470 nm) high frequency stimulation (oHFS, 100 Hz for 1 s, repeated 5 times with 20 s intervals), similar with the LTP induction protocol used in our previous in vivo works in ACC [17], was then delivered to the ACC to activate ChR2-expressing projecting fibers.

We found oHFS induction decreased the mechanical threshold in both sham and SNI mice (Fig. 4C), suggesting that activation of MD-ACC projecting fibers induces mechanical allodynia in sham mice and hyperalgesia in SNI mice. We then observed the possible changed activities of pyramidal cells and interneurons in the ACC. 437 well-isolated neurons in pre- and post-oHFS periods were recognized out of the 442 total recorded units. The internal-spiking interval (ISI) and waveform characteristics of the isolated neurons in each channel were assessed to ensure the two units in pre- and post-HFS periods were from the same neuron (Fig. 4D). We found that, after oHFS induction, the mean firing rate of pyramidal neurons in both the SNI group and sham group increased significantly (Fig. 4E). Meanwhile, the percentage of pyramidal cells with increased firing rate was significantly larger than that with decreased firing rate. In the sham group, the firing rate of 23% pyramidal cells increased, with 10% pyramidal cells decreased and 67% pyramidal cells remained unchanged. In SNI group, the activities of 52%, 9% and 39% pyramidal cells increased, decreased or remained unchanged, respectively (Fig. 4F). However, the mean firing rate of interneurons remained unchanged after oHFS induction in either sham or SNI groups (Fig. 4G). The percentage of interneurons with increased firing rate is also similar with that with decreased firing rate. In the sham group, the firing rate of 30% interneurons increased, with 32% interneurons decreased and 38% interneurons remained unchanged. In SNI group, the activities of 28%, 28%, or 44% interneurons increased, decreased or remained unchanged, respectively (Fig. 4H). These results show that only the activities of pyramidal cells but not interneurons are potentiated by oHFS induction. The potentiated activities of pyramidal cells may account for the behavioral pain hypersensitivity.

## Discussion

In the present study, by mainly using in vivo single-unit recording technique, we showed that it was the increased activity of pyramidal cells but not interneurons in the ACC subject to neuropathic pain. This was also confirmed by morphological results showing that the increased FOS expression induced by neuropathic pain was limited in pyramidal cells but not in interneurons in the ACC. It’s possible that the increased activity of pyramidal cells might be the key factor for the imbalanced E/I ratio in the ACC and for the process of neuropathic pain.

The balance between excitation and inhibition is important for normal brain function and can be disrupted in different pathological conditions like chronic pain. It’s confirmed by different studies that the activity of glutamatergic pyramidal cells in sensory cortex is increased after chronic pain [3, 18]. However, it’s unclear whether the inhibitory activity is accordingly changed. By using in vivo two-photon Ca^2+^ imaging methods, Eto et al*.* find that sensory stimulation induced Ca^2+^ responses of both pyramidal cells and interneurons in somatosensory cortex increase in mice with CFA-induced inflammatory pain [10]. On the contrary, different studies report that local inhibitory synaptic transmissions are decreased in the ACC in mice with bee venom or CFA injection, indicating a decreased GABAergic activity [11–13]. In our present study, we found that the firing rate of ACC pyramidal cells but not interneurons increased in mice with neuropathic pain. The enhanced excitatory activity, without receiving synchronized increased inhibition from local GABAergic interneurons, will significantly damage the E/I balance and lead to a global over-excitation of ACC.

FOS protein is a widely used marker for detecting the neuronal activity [17, 19]. Although the increased expression of FOS has been confirmed in the ACC in mice with neuropathic pain [20, 21], it is not clear whether the increased FOS proteins are co-localized with interneurons. Recently, Shao et al. show that, in rats one month after CFA injection, the increased FOS proteins are only expressed in pyramidal cells but not in interneurons in the ACC [13]. This is in consistent with our results. However, it’s less possible that the interneurons are not involved in nociception regulation, since optogenetic activation [9] or chemogenetic inhibition [13] of interneurons in the ACC reduces mechanical pain responses or deteriorates inflammatory pain-induced anxiety-like behaviors, respectively. We thus propose that the function of ACC interneurons in the regulation of chronic pain might be complicated. The more plausible explanation might be that the interneurons play roles in the early phase of chronic pain or their phasic but not tonic inhibition might be important for the regulation of the global activity of ACC.

LTP is considered as the cellular mechanism of chronic pain in both spinal cord and sensory cortex [18, 22]. In our previous works, we have shown that neuropathic pain occludes the long-term potentiation (LTP) of the synaptic transmission of ACC neurons and inhibition of ACC LTP alleviates the pain responses, indicating that chronic pain induces LTP-like responses of ACC neurons [20, 21, 23]. In the present study, after application of oHFS induction of the ACC afferent fibers containing ChR2, we found that the firing rate of pyramidal cells was increased, suggesting an LTP-like phenomenon. However, unlike the in vitro results in which LTP is occluded in mice with neuropathic pain [21], in vivo oHFS induction also increased the averaged firing rate of pyramidal cells in SNI mice. Furthermore, oHFS induced mechanical hypersensitivity in both sham and SNI group. We thus propose that LTP induction of ACC pyramidal cells should be different in in vivo and in vitro conditions and the potentiated activities of sensory cortical pyramidal cells should further deteriorate the pain responses in mice with chronic pain. Finally, oHFS potentiated the activities of interneurons neither in sham nor in SNI groups. This can be explained as GABAergic interneurons might be less sensitive to HFS induction or interneurons might not be involved in the neuropathic pain-induced hyperactivity of ACC.

In summary, we identified two groups of the main neuronal types, the pyramidal cells and interneurons, in the ACC by using in vivo multichannel recording system. The activities of pyramidal cells but not interneurons are potentiated in mice with neuropathic pain or LTP-like induction. The balance between excitation and inhibition is thus weighed toward excessive excitation in neuropathic pain condition. However, the role of cortical interneurons in the development of chronic pain should be carefully investigated in the future studies.

## Materials and methods

### Animals

The experimental timeline of the study is shown in Fig. 1A. Male C57BL/6J mice (aged 6–8 weeks, weighing 22–26 g) were acquired from the Experimental Animal Center of Fourth Military Medical University. The animals were housed in the laboratory under controlled conditions (temperature: 22–26 ℃, humidity: 40%, light/dark cycle: lights on 9 a.m.-9 p.m.) with food and water available ad libitum. After one-week acclimatization, mice were randomly divided into two groups with six mice each. All operations and handling follow the guidelines of the Fourth Military Medical University Ethics Committee.

### Neuropathic pain model

The spared nerve injury (SNI) model for neuropathic pain was established as described in our previous study [24]. After mice were anesthetized by intraperitoneal injection of 2% pentobarbital sodium (5 mL/kg), three terminal branches of the left sciatic nerve were exposed by direct incision of the skin and a section of the biceps femoris muscle in the left thigh. The tibial nerve and the common peroneal nerve were ligated with 6-0 silk sutures and sectioned distal to the ligation as shown in Fig. 1B. After the ligating and cutting, the nerve was put back to its original position and the muscle and skin were sutured in two layers. In the sham operation group, three branches of sciatic nerve were exposed successively and then disinfected and sutured again, with the nerves not being lesioned.

### Pain behavior tests

Measurements were based on previous reports [22]. Mice were habituated to the testing environment for 3 days before baseline testing and then placed under inverted plastic boxes (7 × 7 × 10 cm) on an elevated mesh floor and allowed to habituate for 30 min before threshold testing. A logarithmic series of 8 calibrated Semmes–Weinstein monofilaments (von Frey hairs; Stoelting, Kiel, WI, USA) (0.008, 0.02, 0.04, 0.16, 0.4, 0.6, 1, 1.4, and 2 g) with various bending forces (0.078, 0.196, 0.392, 1.568, 3.92, 5.88, 9.8, 13.72, and 19.6 mN) was applied to the plantar surface of the hind paw until the mice withdrew from the stimulus. Positive responses included licking, biting, and sudden withdrawal of the hind paws. A von Frey filament was applied 5 times (3 s for each stimulus) to each tested area. The minimum bending force of the von Frey filament able to evoke 3 occurrences of the paw withdrawal reflex was considered the paw withdrawal threshold. All tests were performed in a blinded manner.

Paw withdrawal thermal latency (PWTL) was measured using the UGO BASILE plantar tenderness instrument (Ugo basile, Comerio, Italy) as described in previous study [25]. The mice were put in plastic boxes and adapted to the surrounding environment for 20 min. After that, the radiation light spot of the stimulator was irradiated to the plantar of the mice on the surgical side, and appropriate light stimulation was initiated (the intensity of the beam was adjusted to result in a latency of 8–15 s in Sham mice). Then the latency was measured and recorded every 10 min and repeated for five times. The average value of the results was considered as the threshold of heat pain. In order to avoid tissue damage, the time limit for single irradiation of plantar detection should not exceed 30 s.

The Dynamic mechanical allodynia was tested as described in previous study [26]. The mice were placed on an elevated wire grid and covered with a transparent plastic frame. After 30 min of adapting to the surrounding environment in the frame, the plantar hindpaw was stimulated with a paintbrush from heel to toe. During the test, walking away or occasionally raising the foot score 0, raising the foot for more than 2 s or a single gentle retreat score 1, strongly raising the foot above the body level score 2, and continuously shrinking or licking the foot score 3. Each mouse was measured five times with intervals of 3 min, and the average score of the results was calculated.

### Immunofluorescent histochemical staining

Immunofluorescent histochemical procedure was applied to evaluate the double-labeling of CaMKII/FOS and GAD67/FOS in the ACC of sham and SNI mice. Briefly, mice were perfused with 0.1 mol/L PBS and 4% paraformaldehyde for fixation, and then serially cut into transverse slices with 30 μm thickness. All serial sections were then incubated with primary antisera (1:400, ab11959, Abcam, MA, United Kingdom) for 18–24 h at 4 °C in 0.01 M PBS containing 1% (v/v) normal donkey serum, 0.3% (v/v) Triton X-100, 0.02% (w/v) sodium azide, and 0.12% (w/v) carrageenan (pH 7.4). Then, the sections were incubated with Alexa 488 donkey anti-rabbit (1:500, A21206, Invitrogen)/Alexa 594 donkey anti-mouse (1:500, A21203, Invitrogen, CA), and Alexa 488 donkey anti-mouse (1:500, A21202, Invitrogen)/Alexa 594 donkey anti-rabbit (1:500, A21207, Invitrogen)) for 6–8 h at 4 °C. If necessary, the sections were incubated with tertiary antisera for 2–4 h in 0.01 m PBS with 0.3% (v/v) Triton X-100 at 4 °C. After the immunofluorescence histochemical staining, the sections were observed and images were captured using VS200 microscope (VS200, Olympus, Japan). Digital images were captured using VS200 software (Olympus). Image J (NIH Image) software was utilized to count the number of double labeled neurons using.

### In vivo single-unit recording

In vivo single-unit recordings were performed as previous studies with minor modifications [27, 28]. The four guide tubes contained 16-channel electrodes using 25.4-μm formvar-insulated nichrome wire (Cat No. 761500, A-M System, USA) and a 62.5-μm diameter optical multimode fiber in the center. As shown in Fig. 3A, rAAV-CaMKIIα-hChR2 (E123T/T159C)-mCherry was injected into right MD (ipsilateral to recorded ACC) (1.7 mm posterior to the bregma, 0.3 mm lateral to the midline and 3.43 mm vertical to the skull surface). After 2 weeks, the electrodes/optrode implantation was performed to the right ACC at the following stereotaxic coordinates: 1.1 mm anterior to the bregma, 0.3 mm lateral to the midline and 1.8 mm vertical to the skull surface. Head-fixation was utilized immediately after the implantation of electrode in the ACC, dental adhesive resin cement (Super-bond C&B, Japan) was used to stick the metal head bar to the exposed skull, to ensure the security of the head-fixed position when the head bar was held firmly by the behavioral apparatus during recording [29]. The mice were given at least one week to recover after the implantation of the electrodes. Before recording, mice were habituated to the head-fixed setup for at least 4 sessions (15 min per session, twice/day). To activate MD-ACC projecting fibers, A series of high frequency opto-stimulation (100 Hz for 1 s, repeated 5 times with 20 s intervals) was applied by using a blue laser (470 nm, Inper Ltd, China) after 20 min baseline recording. Recording was then continued for 30 min after HFS induction. Single-unit recording were performed with an Neurostudio System (Jiangsu Brain Medical Technology Co. ltd, China).

### Spike sorting and cell type identification

Single-unit spike sorting was performed by MClust-v4.4 toolbox with MATLAB software (MathWorks, USA). To be precise, based on amplitude and waveform energy features, spikes were manually sorted into clusters. A cluster of waveforms was considered as a single neuron if the ratio of its inter-spike intervals (ISI) under 2 ms was < 1%, and the unit quality was quantified by isolation distance (> 20) and L-ratio (< 0.1) [27]. Besides, the two units were considered as a single neuron when the spike time of all the units measured coincided via the cross-correlation comparison. Pyramidal neurons and gamma-aminobutyric acid (GABA) neurons are two main cell types in ACC region. The single neuron was classified as putative pyramidal neuron mainly based on the criteria as described in a previous study [30]: trough-to-peak duration above 430 μs which exhibited long duration action potentials. GABA neurons were identified mainly based on criteria that trough-to-peak duration under 430 μs.

### Statistical analysis

Statistical analysis was done using the GraphPad Prism (GraphPad Software, San Diego, CA, USA). The *p* values were calculated by Wilcoxon rank-sum test, paired and unpaired *t*-tests, one-way ANOVA with Dunnett post hoc analysis. Results were expressed as mean ± SEM. Statistical significance was set at *p* < 0.05.

## Data Availability

The data that support the findings of this study are available from the corresponding author upon reasonable request.
